# Focal pulsed field electroporation of left ventricular premature contractions after failed radiofrequency ablation

**DOI:** 10.1016/j.hrcr.2023.05.019

**Published:** 2023-06-03

**Authors:** Jim Hansen, Martin A. Haugdal, Arne Johannessen, Morten Lock Hansen, Rene Worck, Martin H. Ruwald

**Affiliations:** Division of Electrophysiology, Department of Cardiology, Herlev and Gentofte Hospital, Hellerup, Denmark

**Keywords:** Electroporation, Pulsed field ablation, Ventricular arrhythmia, Premature ventricular contractions, 3D mapping, Electrogram

## Introduction

Pulsed field electroporation (PFE) has earned substantial attention owing to its unique, non–thermal tissue–preferential mechanism for cardiac ablation of arrhythmias and is used increasingly for pulmonary vein isolation.^1^, ^2^, ^3^ Recently, a pulsed electrical field delivery system consisting of a proprietary generator coupled to specified ablation catheters and 3D electroanatomical mapping systems obtained CE approval for pulmonary vein isolation in patients with paroxysmal atrial fibrillation based on results of the ECLIPSE trial.^4^ The utility of this system for mapping and ablation in human ventricular myocardium in the clinical setting of ventricular arrhythmias was recently reported in case form.^5^ In this case we present the first successful use of PFE for focal endocardial ablation of premature contractions from the left ventricle following extensive unsuccessful attempts at radiofrequency ablation (RFA) in the same procedure. Thus, in a direct intraprocedural comparison, focal PFE was superior to RFA, indicating a promising role for this ablation modality in the future treatment of ventricular arrhythmias.

**Key Teaching Points**

• Catheter ablation of premature ventricular contractions (PVCs) from the basal left ventricle can be difficult in cases of epicardial/deep intramural foci or difficult catheter positioning.
• Radiofrequency ablation (RFA) may not achieve sufficient lesion depth in these cases.
• Focal pulsed field electroporation (PFE) is a new ablation modality that is myocardial tissue preferential, is less dependent on catheter–tissue contact force, and has the potential for greater tissue penetration.
• In the present case, PVCs originating from the superolateral ventricular aspect of the mitral annulus could not be ablated with extensive radiofrequency delivery from the endocardium and via the distal coronary sinus, but was effectively eradicated when the ablative energy was switched to focal PFE from the same endocardial site.
• In a direct intraprocedural comparison, focal PFE was much more efficient than RFA for ablation of PVCs, suggesting a role for this ablation modality in the future treatment of ventricular arrhythmias.

## Case report

**Figure 1:**
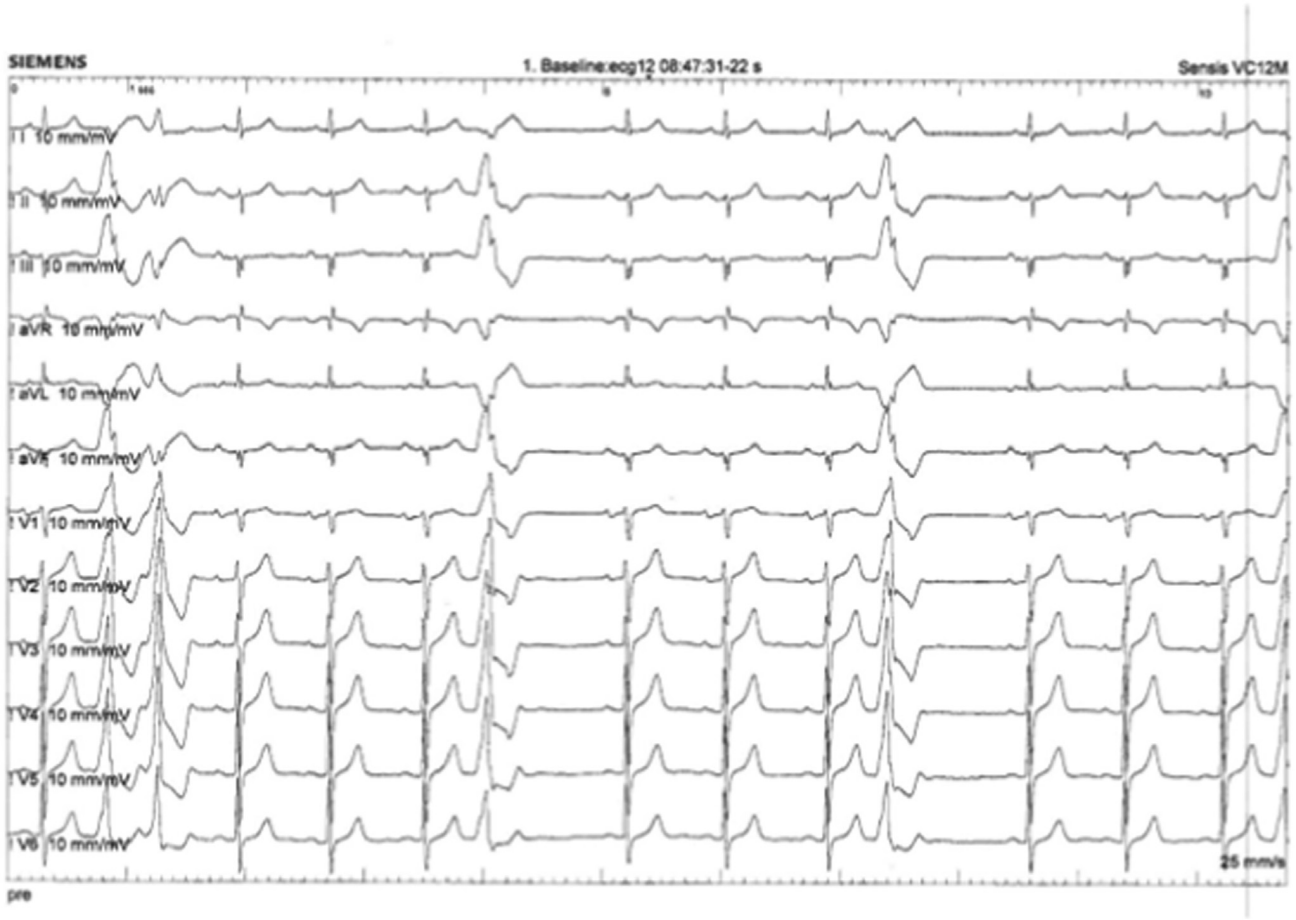
Twelve-lead electrocardiogram (ECG-12) with clinical premature ventricular contractions. ECG-12 showed frequent monomorphic premature ventricular contractions with a QRS morphology suggestive of a superolateral mitral annulus origin.

A 68-year-old male patient with no prior history of cardiovascular disease presented with palpitations and dyspnea. Echocardiography showed a normal ejection fraction of 58%, mild mitral regurgitation, and enlarged left atrium. Coronary angiography by computed tomography was normal. Twelve-lead electrocardiography (ECG-12) was normal, but showed frequent monomorphic premature ventricular contractions (PVCs) with a QRS morphology suggestive of a superolateral mitral annulus origin (Figure 1). On Holter monitoring PVC burden was 14%, including bigemini, pairs, and triplets. The patient was unsuccessfully tried on beta-blockers and subsequently referred for catheter ablation.

### Management

**Figure 2:**
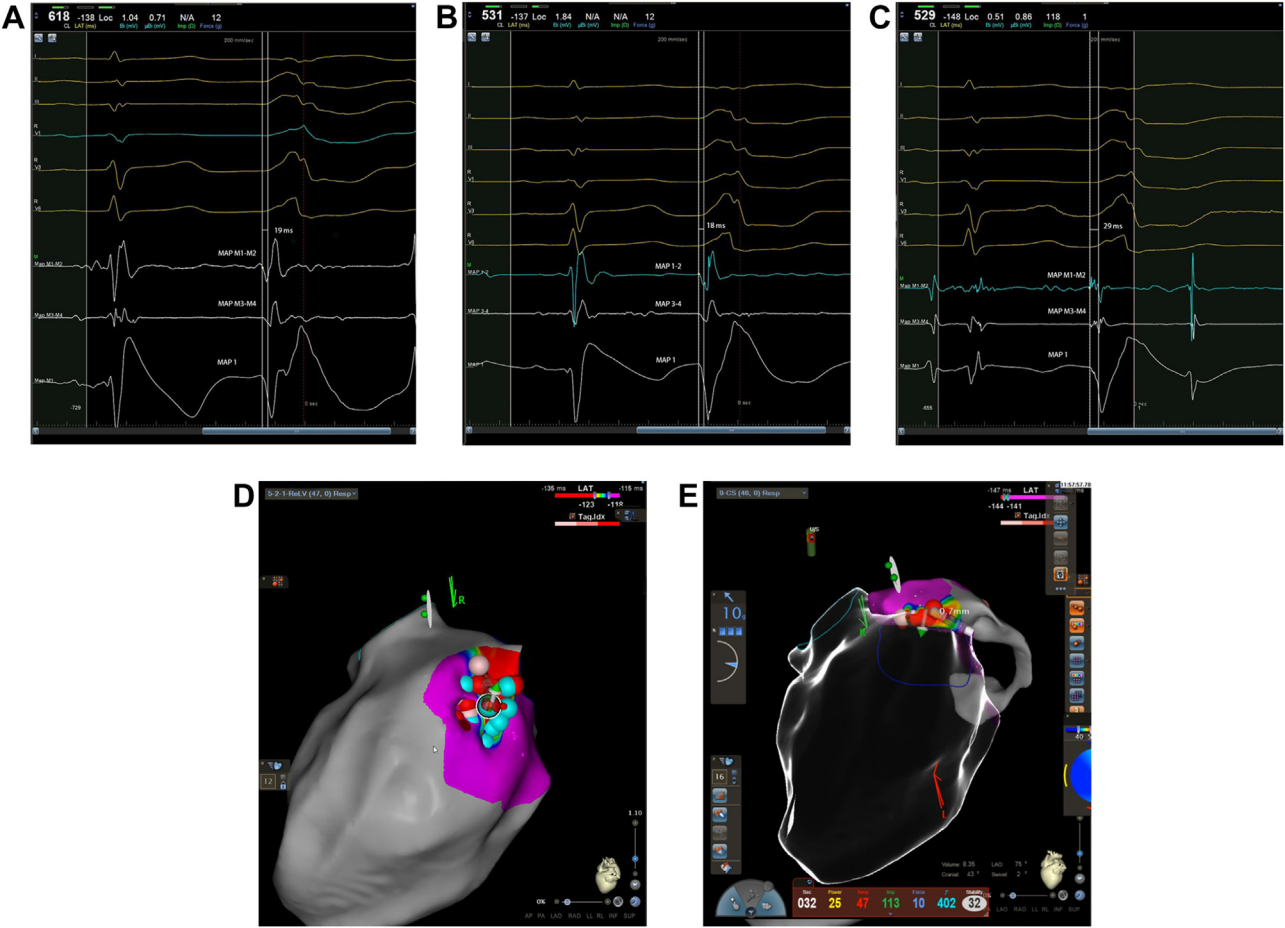
Earliest activation electrogram and anatomical ablation sites endocardially and from the distal coronary sinus. **A:** Earliest endocardial activation was found on the ventricular aspect of the mitral annulus in a left anterior oblique view 1-o’clock position, which preceded QRS onset by 19 ms recorded with the radiofrequency (RF) ablation catheter. **B:** Earliest endocardial activation recorded with the pulsed field electroporation (PFE) delivery catheter preceding QRS onset by 18 ms. **C:** Earliest activation in the distal coronary sinus, which preceded QRS onset by 29 ms. **D:** Ablation sites and catheter placement from the endocardial approach. **E:** The corresponding ablation site in the great cardiac vein – distal coronary sinus. Red tags represent RF ablation sites and blue tags represent PFE delivery sites.

**Figure 3:**
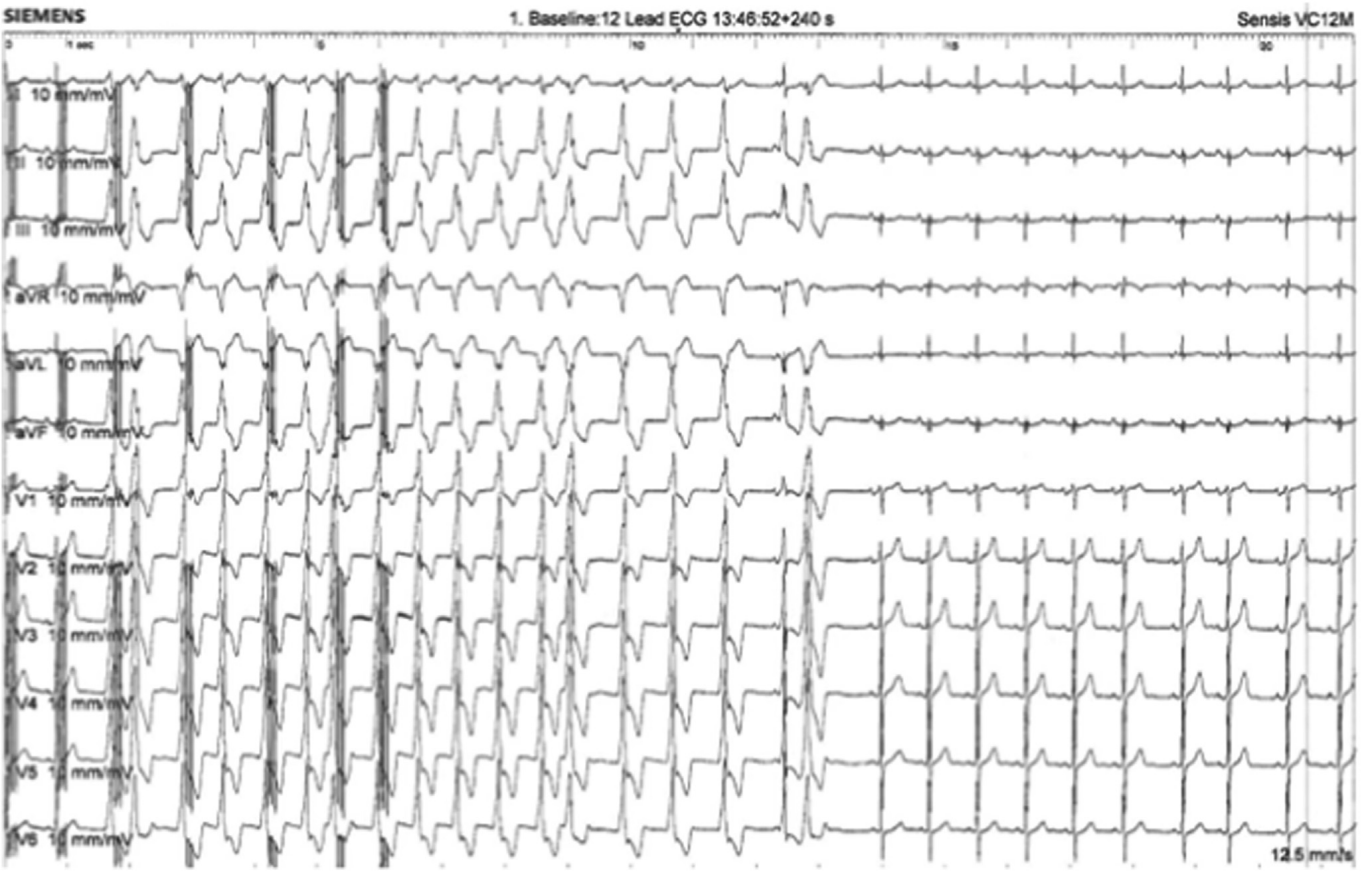
Excitatory response to pulsed field electroporation delivery on 12-lead electrocardiogram (ECG-12). The figure shows the excitatory premature ventricular contraction response to trains of pulsed field electroporation delivery on ECG-12 and, hereafter, sinus rhythm.

The procedure was scheduled for RFA under light conscious sedation using intracardiac echo for precise ultrasound-guided 3D anatomical mapping of left ventricular structures. The patient had frequent spontaneous monomorphic PVCs during the procedure, allowing for a local activation time (LAT) mapping approach. Vascular access was obtained by placement of 3 sheaths in the right femoral vein and 1 sheath in the right femoral artery, as the need for dual left ventricular access was anticipated. The coronary sinus (CS) was instrumented by a 2F multipolar mapping catheter guided by a large-curl steerable sheath and a guiding catheter, and a 10F intracardiac ultrasound catheter was advanced successively to the right atrium, right ventricle, and CS for generation of focused 3D maps of the basal left ventricle and mitral annulus. Retrograde access to the left ventricle from the right femoral vein was attempted, but had to be abandoned owing to a tortuous course of the iliac artery and abdominal aorta. Instead, the left ventricle was accessed by transseptal puncture, and an irrigated contact force sensing ablation catheter was introduced, supported by a second large-curl steerable sheath. Earliest endocardial LAT was found on the ventricular aspect of the mitral annulus in a left anterior oblique view 1-o’clock position and preceded QRS onset by 19 ms (Figure 2A and 2B). Earliest epicardial LAT was found in the distal CS, proximal to the descent of the anterior interventricular vein, as demonstrated by CS contrast angiography, where LAT preceded QRS onset by 29 ms (Figure 2C). On fluoroscopy, earliest endocardial activation site was <1 cm from earliest epicardial (perivascular) activation; and therefore, by our preference, ablation was attempted from the endocardium. Extensive delivery of radiofrequency (RF) energy in a temperature-controlled mode (35–50 W for up to 120 seconds) caused only temporary suppression of PVCs (Figure 2D). Supplemental RF delivery at the earliest activation site in the CS (25–30 W for up to 90 seconds) (Figure 2E) also had only transient effect, and after a total of 19 minutes of RF delivery the decision was made to switch to PFE as the ablative energy. The set-up was converted to general anesthesia, which had no effect on PVC frequency. The ablation catheter was switched to an irrigated-tip contact force-sensing catheter approved for PFE delivery, and ablation was guided by the previously generated LAT maps. PFE (25-ampere, R wave–synchronized trains of unipolar pulses) was delivered to the site of earliest activation, which preceded QRS onset by 18 ms (Figure 2B), preceded by 0.4 mg nitroglycerine intravenously to prevent coronary vasospasm. In the first PFE delivery at an endocardial site corresponding to previous RFA sites (Figure 2D), a marked excitatory response of PVC activity was noted during and immediately following the application (Figure 3). After this, there were no more PVCs, but additional PFE applications (7 in total) were placed in the circumference of the initial ablation site to secure efficacy. No ST-T segment changes were observed on ECG-12. Heart rhythm was monitored for 40 minutes, including isoprenaline challenge and return to consciousness without PVC recurrence, and the procedure was terminated. A full video case presentation is supplied in Supplemental Video Clip 1.

## Discussion

This case demonstrates an impressive difference in ablative efficiency between focal RFA and focal PFE in the left ventricular myocardium. In the same procedure, with the same operator, targeting the same site, a few applications of focal PFE accomplished what had not been achieved by extensive RFA from this and adjacent sites—elimination of the clinical PVCs.

Based on the precocity of CS electrograms vs earliest endocardial activation and electroanatomical mapping data, we believe this was a case of a deep intramyocardial/epicardial focus that could not be penetrated by RF lesions with the catheter positions available for this procedure. Although PFE delivery in the ventricles is at present off-label use, preclinical work has demonstrated that this system with unipolar PFE at a 25-ampere setting can deliver lesions of more than 8 mm depth in porcine myocardium.^6^ Thus, we decided to attempt this approach to produce more efficient lesions, and the outcome suggests this was indeed achieved. A number of safety concerns had to be considered. First, delivery of high-voltage pulses to the ventricles could trigger arrhythmias. However, the generator delivers pulsed fields synchronized to the R wave, which in preclinical work has been shown not to trigger ventricular arrhythmias.^7^ Second, PFE delivery with a different system used off label in the anterior right or left atrium has recently been shown to potentially induce vasospasm in adjacent coronary arteries, but this adverse effect could be prevented by pretreatment with intravenous nitroglycerine.^8^ Based on these findings, and on 2 other reported clinical cases using PFE by another system for ventricular arrhythmias without adverse events,^9^^,^^10^ it was deemed safe to perform focal PFE in the present case, and none of the potential adverse effects were observed.

Because PFE was performed after RFA in this procedure, we need to consider a potential contribution of pretreatment with RFA at the ablation site. However, recent preclinical work suggests PFE penetration in mature scar (including RFA-generated) is not different from normal myocardium.^7^ Although the effects of acute RFA-induced changes prior to PFE cannot be excluded, we believe it to be an unlikely explanation for the marked effect of PFE seen in this case, but clearly more work is needed to investigate pulsed electrical field design and catheter delivery for optimal lesion depth and safety in healthy and diseased ventricles.^11^

### Follow-up

The patient was discharged the next day without a single PVC on continuous monitoring. When contacted 1 week after the procedure, he reported to have no palpitations. The patient experienced recurrent palpitations weeks after the procedure; on XYZ Holter this was found to be owing to 4% PVCs from a different focus from the one treated, as well as premature atrial contractions and atrial tachycardia. There was no recurrence of the clinical PVC ablated in the case.

## Conclusion

The unique insight from this case indicates that focal PFE can be much more efficient than RFA for ablation of PVCs. It remains to be seen if the safety and efficacy off this off-label use of focal PFE can be confirmed in systematic studies

## Appendix Supplementary Data

Supplemental Video Clip 1 This video shows the full case presentation starting with pre-ablation 12- lead ECG, the PVC location, the timing of intracardial electrograms to the earliest activation with ablation targets and ablation tags for both radiofrequency ablation and later pulsed field electroporation with 12-lead ECG excitatory response of PVCs and termination.
